# IP4GS: Bringing genomic selection analysis to breeders

**DOI:** 10.3389/fpls.2023.1131493

**Published:** 2023-03-06

**Authors:** Tong Li, Shan Jiang, Ran Fu, Xiangfeng Wang, Qian Cheng, Shuqin Jiang

**Affiliations:** Frontiers Science Center for Molecular Design Breeding, College of Agriculture and Biotechnology, China Agricultural University, Beijing, China

**Keywords:** bioinformatics, genomic selection, genotype-to-phenotype prediction, web-based platform, R shiny

## Abstract

Genomic selection (GS), a strategy to use genotypes to predict phenotypes *via* statistical or machine learning models, has become a routine practice in plant breeding programs. GS can speed up the genetic gain by reducing phenotyping costs and/or shortening the breeding cycles. GS analysis is complicated involving data clean up and formatting, training and test population analysis, model selection and evaluation, and parameter optimization. In addition, GS analysis also requires some programming skills and knowledge of statistical modeling. Thus, we need a more practical GS tools for breeders. To alleviate this difficulty, we developed the web-based platform IP4GS (https://ngdc.cncb.ac.cn/ip4gs/), which offers a user-friendly interface to perform GS analysis simply through point-and-click actions. IP4GS currently includes seven commonly used models, eleven evaluation metrics, and visualization modules, offering great convenience for plant breeders with limited bioinformatics knowledge to apply GS analysis.

## Introduction

Polygenic traits of plants, such as grain yield (GY), flowering time (FT), and plant height (PH), are usually controlled by many minor effect genes. In such cases, traditional marker-assisted selection (MAS), which relies on statistical power to identify makers/genes-traits associations, cannot effectively be applied to expedite trait improvement of polygenic traits. (Collard and Mackill, 2008; Xu and Crouch, 2008). Genomic selection (GS) predicting phenotypes from genome-wide molecular markers, may act as a complementary approach for the improvements of polygenic traits. (Desta and Ortiz, 2014; Crossa et al., 2017). GS models use genome-wide genetic markers to predict phenotypes, which can maximumly capture phenotypic variation contributed by multiple minor effect genes. (Cerrudo et al., 2018). To perform GS analysis, first, a GS model is built by genotypic and phenotypic data from a training population. The model is then employed to predict phenotypes of a candidate population based on their genotypic data. (Meuwissen et al., 2001; Desta and Ortiz, 2014). The advent of advanced next-generation sequence technologies, e.g. genotyping by targeted sequencing (GBTS) has significantly reduced the cost of genotyping. (Guo et al., 2019). Wide application of GS to more and more plant species has become feasible since genotyping cost is no longer a bottleneck (Bauck et al., 2018; Belamkar et al., 2018; Atanda et al., 2021; Wang et al., 2021).

Most popularly GS models are linear regression (or parametric) regression models. These models include the best linear unbiased prediction (BLUP) method represented by RRBLUP (ridge regression BLUP) and the Bayesian method represented by BayesA and BayesB (Meuwissen et al., 2001; Endelman, 2011). In recent years, machine learning (ML)-based methods have been introduced to build GS models, such as support vector machine (SVM), random forest (RF), deep learning, and light gradient boost (LGB) algorithms (Blondel et al., 2015; Qiu et al., 2016; Ma et al., 2018; Yan et al., 2021). ML methods have been proposed to be better than linear models to incorporate nonlinear relationships between genotypes and phenotypes. However, ML may require relatively larger data size to achieve better performance and outperform linear models on more complex datasets. (González-Recio and Forni, 2011; Yan et al., 2021; Yan and Wang, 2022). In the practice of plant breeding at the current stage, most of the time, the size of training datasets is not big enough and the property of datasets is still relatively simple, because the high cost of phenotyping must still be considered as an important factor. In this situation, BLUP and Bayesian methods outperform most ML methods and have thus gained popularity in plant breeding (Tanaka and Iwata, 2018; Yan et al., 2021).

Among all available GS methods, RRBLUP, a “simple” model that assumes all marker effects possessing the same variance, is the most widely used approach in GS application. It has therefore become the baseline prediction model for the evaluation performance of other GS models. However, the assumption of equal variance of marker effects may be not realistic. Bayesian methods which assumed prior distributions for the variance of marker effects, has become another popular GS approach due to its potential for better estimating of marker effects. Currently, the majority of GS tools are command-line interfaces, requiring advanced data management skills, good programing skills and knowledge of statistical modeling, which restrict the practical use by breeders in the seed industry. There has therefore been an urgent need to develop a web-based platform with a user-friendly interface for facilitating the use of GS strategies among the plant breeding community. To solve this problem, we developed IP4GS, a web-based interactive platform for genomic selection. IP4GS includes seven GS models and eleven evaluation metrics to help users select optimal models for GS analysis. It also includes bioinformatics pipelines for preprocessing of genotypic data and visualization modules for population analysis. The functionality of all modules may be invoked simply by point-and-click actions through a user-friendly interface developed using shiny.

## Methods

### Demo datasets for developing IP4GS

The public dataset from a population of 1,404 maize F1 hybrids, which generated by crossing 1,404 inbred lines with an elite tester line Zheng58, was used to develop the web-based platform of IP4GS (Liu et al., 2020; Xiao et al., 2021). The genotypic data of the 1,404 F_1_ hybrids includes 4,903 SNPs selected from a total of 14.8 million SNPs; details of SNP selection were previously described by Cheng et al. (Cheng et al., 2021). Phenotypes include flowering time (FT), plant height (PH), and grain yield (GY) (Xiao et al., 2021). The demo datasets of genotypes and phenotypes are accessible at https://github.com/furan2019/IP4GSdata.

### Implementation of a web-based IP4GS platform

Shiny is an R-based framework, allowing programmers to develop interactive web applications (https://www.rstudio.com/products/shiny/). Benefitting from the expandability and useability, Shiny has been widely used to develop online applications for bioinformatics software or interactive plot tools (McMurdie and Holmes, 2015; Yu et al., 2018; Sievert, 2020). The shiny-based application can be accessed on the local host or deployed on the public internet for public access. A shiny-based application usually consists of two parts, a user-interface (UI) script (IP4GS_UI.r for IP4GS) and a server script (IP4GS_server.r for IP4GS). The UI script controls the layout of different panels and visualization of results and bridges the user inputs and background functions. IP4GS utilized several packages to enrich and improve the UI interactive experience, such as “DT” for dynamic tables, “plotly” for dynamic plots, “shinycssloaders” for loading animations, “shinybusy” for progress notification, and “shinyWidgets” for input of multiform parameters and dynamic controls. HTML5 language and condition panels were also introduced to improve and optimize the layout of panels. The server script plays an important role in shiny-based applications. All functions provided by IP4GS were achieved by server script, including data input, data preprocessing, and GS model building and evaluation. For real-time interaction, user-defined parameters and operations are passed to the server script, which then executes the corresponding functions and formats the outputs. Lastly, the server script returns the results to a specific location according to flags that can bridge the UI script and server script.

### GS models and evaluation metrics in the GS analysis panel

IP4GS integrates five linear models and two ML methods for GS application, which are all called from R libraries (**Table 1**). The RRBLUP method is called from the package “rrBLUP”. The RRBLUP model is based on the assumption that all markers have equal variance with small and nonzero effect (Endelman, 2011). The three Bayesian methods, BayesA, BayesB, and BayesC, are called from the package “BGLR (Bayesian generalized linear regression)” (de los Campos and Pérez, 2015). BayesA utilizes a scaled *t*-distribution for estimating marker effects. BayesB is similar to BayesA, with the main difference that it utilizes both shrinkage and variable selection algorithms to estimate marker effects. By contrast, BayesC estimates marker effects based on a Gaussian distribution. The LASSO (least absolute shrinkage and selection operator) method is called from the package “glmnet (Lasso and elastic-net regularized generalized linear models),” which combines both shrinkage and variable selection algorithms to estimate marker effects. The two ML methods, SVR (support vector regression) and RFR (random forest regression), are called from the “e1071” and “randomForest” packages, respectively. SVR finds an appropriate line (or hyperplane in higher dimensions) to fit the data, and RFR uses a regression model rooted in bootstrapping sample observations (Liaw and Wiener, 2002; Meyer et al., 2014; Ornella et al., 2014).

**Table 1:** Seven GS methods integrated into IP4GS.

Model	Important parameter (s)	Package	Reference
BayesA	nlter burnIn thin	BGLR	(de los Campos and Pérez, 2015)
BayesB			
BayesC			
Least absolute shrinkage and selection operator (LASSO)	alpha	glmnet	(Friedman et al., 2010)
Ridge regression best linear unbiased prediction (RRBLUP)	N/A	rrBLUP	(Endelman, 2011)
Support vector regression (SVR)	gamma; cost; kernel	e1071	(Meyer et al., 2014)
Random forest regression (RFR)	ntree; nodesize	randomForest	(Liaw and Wiener, 2002)

To enable comprehensive evaluation of the prediction accuracy of selected GS models, IP4GS integrates eleven evaluation metrics: five correlation-based metrics to globally measure the relationship between observed and predicted phenotypes, and six threshold-based metrics to count accurately predicted top-ranked individuals (**Table 2**). The correlation-based methods are Pearson correlation coefficient (PCC), Kendall rank correlation coefficient (KCC), Spearman rank correlation coefficient (SCC), squared *R* coefficient of determination (*R*^2^), and mean squared error (MSE). These metrics usually measure the global performance of models. For example, KCC treats all pairs equally; however, in breeding practice, more attention should be paid to extreme values such as high yield and short flowering time (Blondel et al., 2015). Thus, six threshold-based metrics were introduced for top-*k* individuals with ideal phenotypic value. These are normalized discounted cumulative gain (NDCG), mean NDCG, relative efficiency (RE), Accuracy, *F*-score, and Cohen’s kappa coefficient (Kappa) (Ornella et al., 2014; Blondel et al., 2015). The calculation methods for these metrics are described in **Table 2**. *X* is an array of observed phenotypic values, and *Y* is an array of predicted phenotypic values. For Accuracy, *F*-score, and Kappa, positive samples are those with ideal phenotypic value.

**Table 2:** Eleven metrics for model evaluation integrated into IP4GS.

Evaluation Metric	Formula	Remarks
Pearson correlation coefficient (PCC, *r*, *R*)	$\text{PCC}\left(X,Y\right)=\;\frac{\sum_{i=1}^{n}\left(x_{i}-\bar{x}\right)\left(y_{i}-\bar{y}\right)}{\sqrt{\sum_{\text{i}=1}^{n}{\left(x_{i}-\bar{x}\right)}^{2}}\sqrt{\sum_{i=1}^{n}{\left(y_{i}-\bar{y}\right)}^{2}}}$	$\bar{x}$ is the mean of *X*, $\bar{y}$ is the mean of *Y*
Kendall rank correlation coefficient (KCC, tau, τ)	$\text{KCC}\;\left(X,Y\right)=\;\frac{N1-N2}{n\left(n-1\right)/2}$	*N*1: number of concordant pairs (e.g., *x*_*i*_ and *y*_*i*_); *N*2: number of discordant pairs
Spearman rank correlation coefficient (SCC, rho, ρ)	$\text{SCC}\left(X,Y\right)=\;\frac{\text{cov}\left(R\left(X\right),R\left(Y\right)\right)}{\sigma_{R\left(X\right)}\sigma_{R\left(Y\right)}}$	*R*(*X*) and *R*(*Y*) are the rank of variables
Coefficient of determination, *R* squared (*R*^2^, *r*^2^)	$R2\;\left(X,\;Y\right)=\;1-\;\frac{\sum_{i=1}^{n}{\left(x_{i}-y_{i}\right)}^{2}}{\sum_{i=1}^{n}{\left(x_{i}-\bar{x}\right)}^{2}}$	$\bar{x}$ is the mean of *X*
Mean squared error (MSE)	$\text{MSE}\left(X,Y\right)\;=\;\frac{\sum_{i=1}^{n}{\left(x_{i}-y_{i}\right)}^{2}}{N}$	*N* is the total number of samples
Normalized discounted cumulative gain (NDCG)	$\text{NDCG}\left(k,\;X,\;Y\right)=\;\frac{\sum_{i=1}^{k}x\left(i,Y\right)d\left(i\right)}{\sum_{i=1}^{k}x\left(i,X\right)d\left(i\right)}$	$d\left(i\right)=\;\frac{1}{\log_{2}i+1}$ is a discount function; *x*(*i*,*Y*) is the *i*th value of *X* with the order of *Y; x*(*i*,*X*) is the *i*th value of X with the order of *X*; *k* is the top *k* individuals with ideal phenotypic values; $\bar{x}$ is the mean of *X*
Mean normalized discounted cumulative gain (mean NDCG)	$\text{meanNDCG}@K\left(X,Y\right)=\;\frac{1}{K}\sum_{k=1}^{K}\text{NDCG}\left(k,X,Y\right)$	
Relative efficiency (RE)	$\text{RE}\left(k\right)=\;\frac{\;\frac{1}{k}\sum_{i=1}^{k}x\left(i,Y\right)-\bar{x}}{\frac{1}{k}\sum_{i=1}^{k}x\left(i,X\right)-\bar{x}}$	
Accuracy	$\text{Accuracy}=\;\frac{N_{\text{TP}}+N_{\text{TN}}}{N}$	TP: true positive; TN: true negative; FP: false positive; FN: false negative; default *β* = 1; $P=\frac{N_{\text{TP}}}{N_{Op}\;};R=\frac{N_{\text{TP}}}{N_{Pp}\;}$ ; *N_Op_ = N*_TP_ *+ N*_FN_; *N_On_ = N*_FP_ *+ N*_TN_; *N_Pp_ = N*_TP_ *+ N*_FN_*; N_Pn_ = N*_FN_ *+ N*_TN_.
*F*-score	$F_{\beta }-\text{score}=\;\frac{\left(1+\beta^{2}\right)\times P\times R}{\beta^{2}\times P+\;R}$	
Cohen’s kappa coefficient (Kappa)	$\text{Kappa}=\;\frac{p_{0}-p_{e}}{1-p_{e}}$ ; $p_{0}=\;\frac{{\text{N}}_{\text{TP}}+\;{\text{N}}_{\text{TN}}}{N}$ ; $p_{e}=\frac{N_{Pp}}{N}\;\times \;\frac{N_{Op}}{N}+\;\frac{N_{Pn}}{N}\;\times \;\frac{N_{On}}{N}$	

### Bioinformatics pipelines in the data preprocessing panel

Currently, IP4GS accepted genotypes with AA/AB/BB alleles (allele format) and 0/1/2 format (numeric format). When data processing button is pressed, the data processing function will compute allele frequency, define the major and minor alleles, filter and format submitted genotypic data. It is worth noting that genotypic data in numeric format is not applicable to this function, IP4GS suppose that genotypic data submitted in numeric format is already processed by users. For genotypic data in

allele format, the function will compute allele frequency and missing rate, define the major and minor alleles. Genotypic data will be filtered by minor allele frequency (MAF) and missing rate, markers with MAF below 0.05 (<= 0.05) or missing rate higher than 0.2 (>= 0.2) will be removed and the criteria can be defined by users. For format conversion, IP4GS uses a common 0, 1, 2 coding scheme based on defined major and minor alleles, AA (homozygous genotype comprising major alleles), AB (homozygous genotype) and BB (homozygous genotype comprising minor alleles) will be coded as 0, 1, and 2, respectively. Two methods are provided in IP4GS for imputation of missing genotype values, “Mean” for mean value of each SNP and “Major” for the code with highest frequency of each SNP. In addition, IP4GS can implement dimensionality reduction of genotypic data using three algorithms, the “prcomp” function with default parameters (e.g., center = TRUE and scale. = FLASE) in the “stats” package for principal component analysis (PCA), the “umap” package for uniform manifold approximation and projection (UMAP), and the “tsne” package for *t*-distributed stochastic neighbor embedding (*t*-SNE). For current version of IP4GS, the utilization of all above three algorithms with default parameters except the dimension which can be defined by users.

### Permissions and accessibility

The free version of IP4GS for academia distributed in the public domain is available at https://ngdc.cncb.ac.cn/ip4gs/. All functions of the public IP4GS described herein are freely accessible for small datasets. There are suggested limitations on the number of SNP markers (<10,000 SNPs) and samples (<1,000 individuals) because of the limited high-performance computing resources of the public web server hosting IP4GS. Considering the need for confidentiality of breeding data by industrial users, an offline version of IP4GS that can be installed on a private server or local devices without any limitation on the number of SNPs and samples is also available. Users interested in nonlimited IP4GS may contact the corresponding author for access.

## Results

### Overall workflow

**Figure 1:**
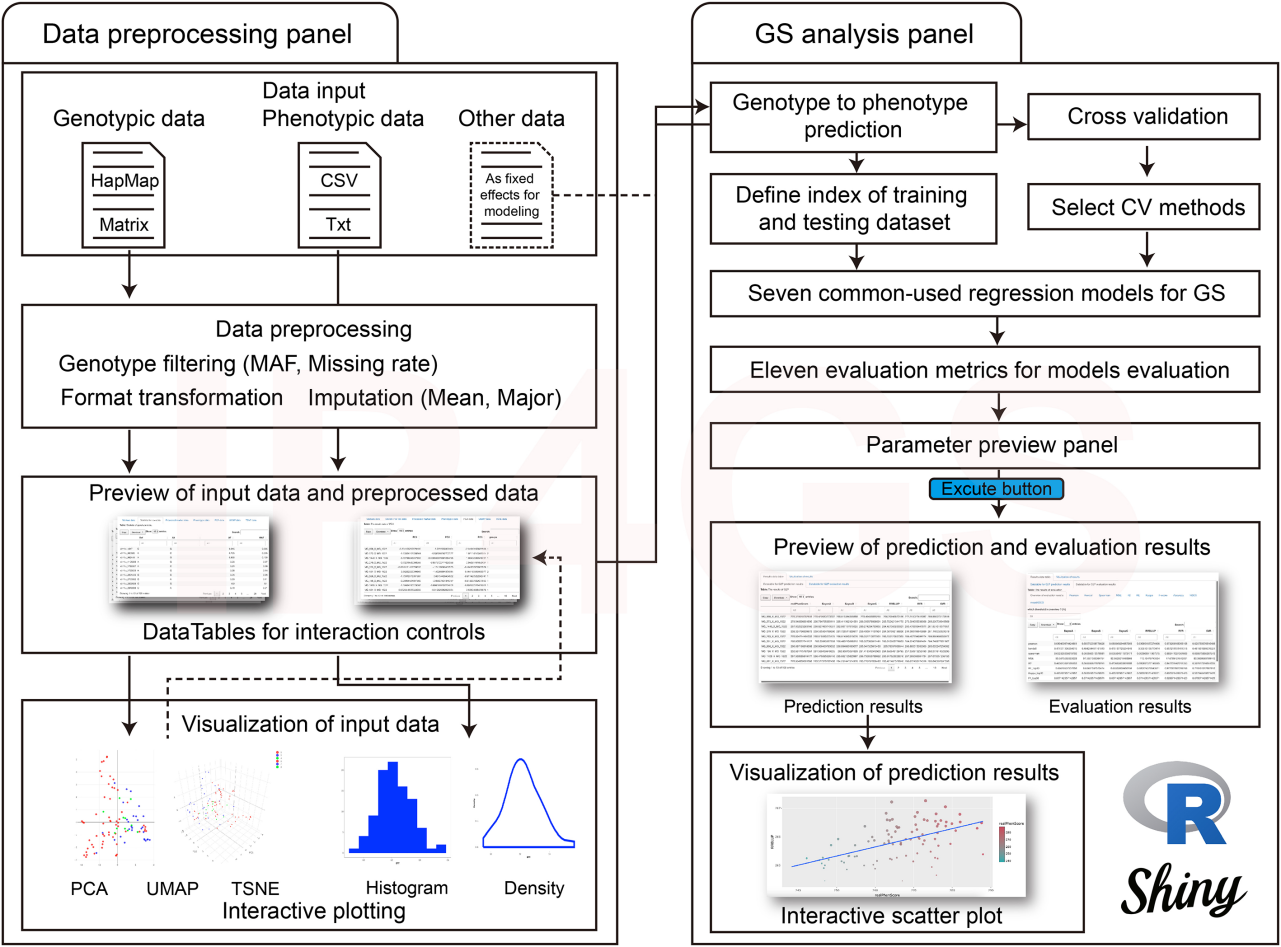
Overall workflow of IP4GS. IP4GS comprises two panels of functional modules: the Data preprocessing panel (left) and the GS analysis panel (right).

The IP4GS platform can be divided into two main panels of functional modules: the “Data preprocessing” panel and the “GS analysis” panel. The Data preprocessing panel comprises not only bioinformatics pipelines for data processing and quality control of input datasets, but also a variety of dimensionality reduction (DR) algorithms for population structure visualization based on genotypes (**Figure 1**, left). Additionally, users may either preview the processed data through the web browser or download the data to a local computer. The GS analysis panel takes the input datasets generated from the first panel and performs G2P prediction with seven regression-based GS models and eleven evaluation metrics (**Figure 1**, right). The parameters for each GS model can be predefined on the parameter input panel by users. Visualization of evaluation results allows users to identify and output predicted phenotypes from the optimal model.

### Functional modules in the data preprocessing panel

**Figure 2:**
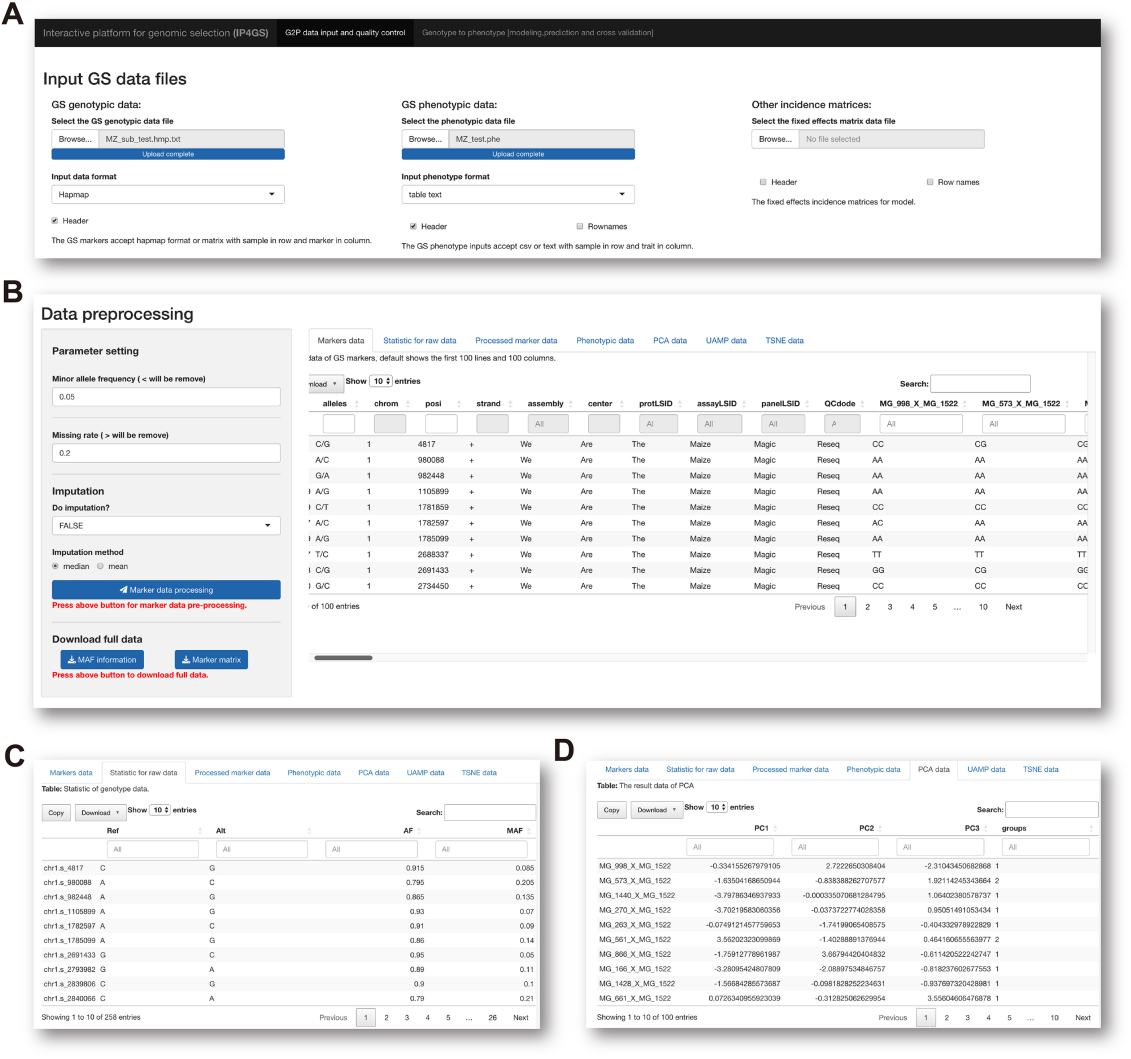
Preprocessing of genotypic data. **(A)** IP4GS accepts genotypic data in HapMap, Matrix, and Custom file formats, phenotypic data in CSV and tab-delimited text formats, and an optional file including features as fixed effects. **(B)** Control console for genotypic data filtration (left) and display window for preview of a variety of data tables (right). **(C)** Preview of processed genotypic data with statistics for MAF, AF, and MR for each SNP. **(D)** Preview of DR data generated by PCA, UMAP, and t-SNE algorithms.

To run the analytical modules in the Data preprocessing panel, users need to prepare one mandatory input file of genotypic and phenotypic data and one optional file containing data for fixed effects considered by the model (**Figure 2A**). Acceptable data formats for the genotypic data file are either the standard “HapMap” format or a plain text file containing a matrix of genotypes in columns and individuals in rows. In the matrix file, the genotype of each SNP must be converted by the users to “0,” “1,” or “2,” representing homozygous major alleles, heterozygous alleles, and homozygous minor alleles, respectively. If users select the “Custom” option from the pulldown list of file formats, the genotype of each SNP can be entered in the character format “A, C, G, T,” which is automatically converted to the “0, 1, 2” format on the basis of allele frequency computed by IP4GS. Either a.csv (comma-separated values) file or a tab-delimited.txt (text) file is acceptable by IP4GS for phenotypic data.

After the input files are uploaded, the genotypic data file is first processed with regard to two criteria, namely minor allele frequency (MAF, default<= 0.05) and missing rate (MR, default >= 0.2), to remove low-quality SNPs (**Figure 2B**). In addition, IP4GS offers users the option of whether imputation is performed on genotypic data or not. As reference haplotype-based imputation may consume a large volume of computing resources, IP4GS only offers a simplified imputation method using “Mean” or “Major” to replace missing genotypic values (see Methods). The preprocessed and filtered genotypic data can then be partially previewed or fully downloaded by clicking the download button in the console (**Figure 2C**). In addition to the raw and processed genotypic and phenotypic data, the DR data computed using PCA (principal component analysis), UMAP (uniform manifold approximation and projection), and *t*-SNE (*t*-distributed stochastic neighbor embedding) algorithms can also be previewed and downloaded (**Figure 2D**).

**Figure 3:**
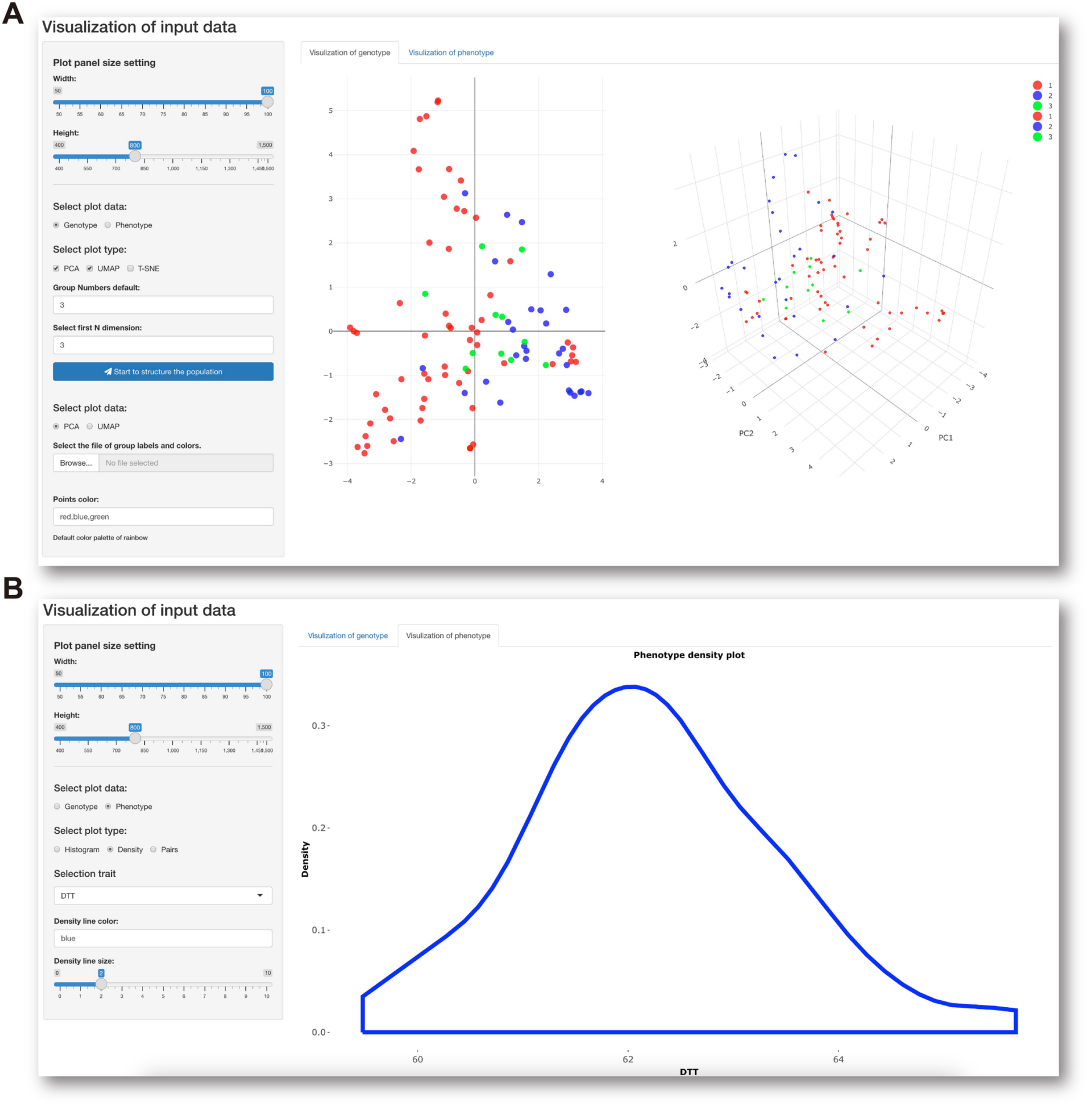
Data visualization console. **(A)** Parameter-setting console (left) for visualization of population structure (right) based on genotypic data. **(B)** Parameter-setting console (left) for visualization of data distribution (right) of selected phenotypic data.

The last module in the Data preprocessing panel is interactive visualization of genotypic and phenotypic data with a control console and display window. This function facilitates not only visualization of the genetic composition of the population subjected to GS analysis (**Figure 3A**) but also understanding of the distribution of phenotypes about to be predicted (**Figure 3B**). When all data preprocessing is complete, IP4GS performs a quality-control assessment on the processed data to ensure proper execution of the subsequent GS analysis.

### Functional modules in the GS analysis panel

After the completion of quality control, users may proceed to the GS analysis panel. The current version of IP4GS supports seven GS methods commonly used in plant breeding: five linear methods (RRBLUP, BayesA, BayesB, BayesC, and LASSO) and two ML methods (the RFR and SVR algorithms) (**Table 1**). We highly recommend that users try all seven methods for initial evaluation of G2P prediction results for a given set of data since it has previously been reported that no single GS method is superior for all traits and species (Ornella et al., 2014; Yan et al., 2021; Robert et al., 2022). It is important to select the optimal model given a designated trait and species to ensure the most precise prediction. Additionally, IP4GS offers eleven evaluation metrics for comprehensive evaluation of model performance; these include not only correlation-based Pearson, Spearman, and Kendall algorithms but also other algorithms such as *F*-score and MSE (**Table 2**).

**Figure 4:**
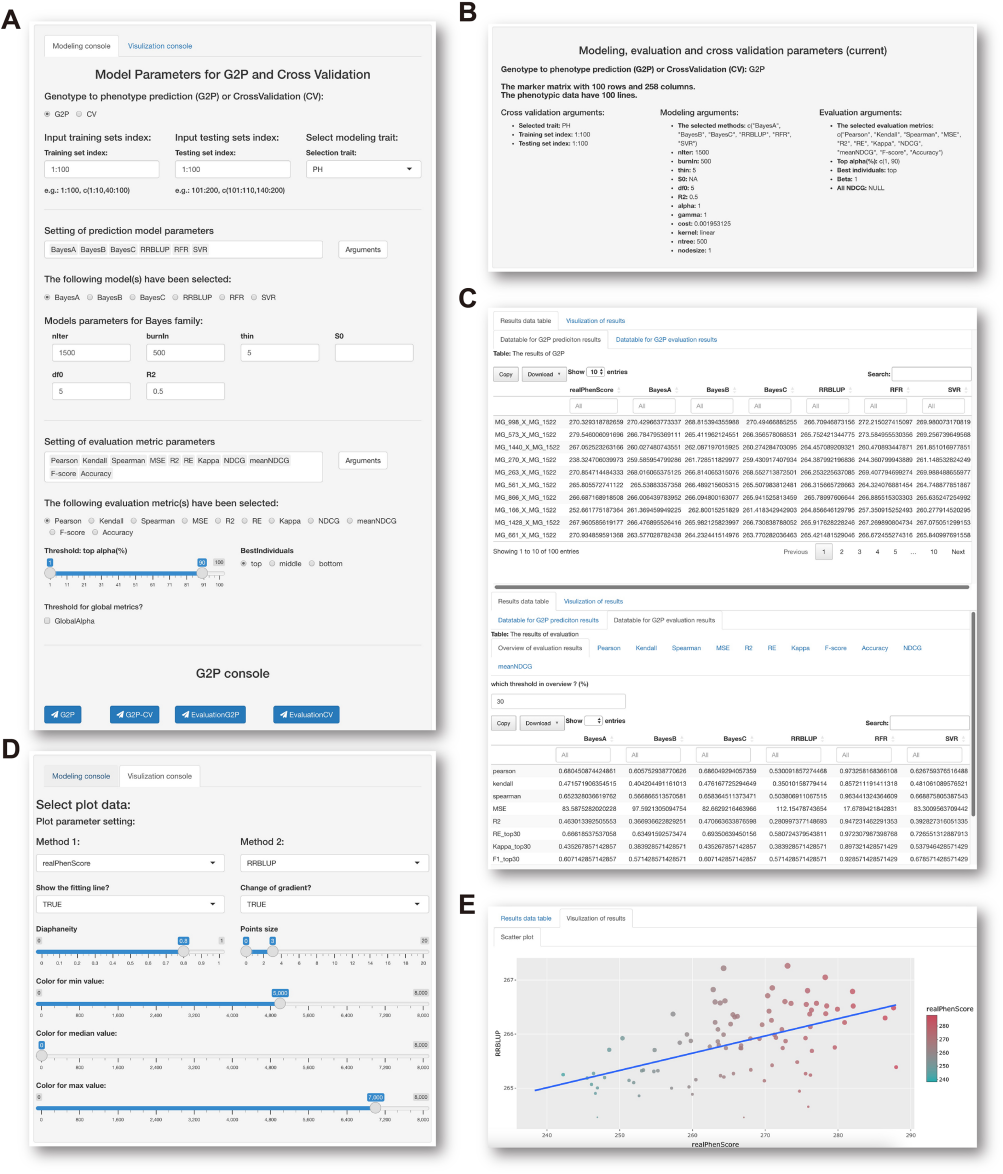
GS analysis panel. **(A)** Modeling console for selection of GS methods, evaluation metrics, and setting of model parameters. **(B)** Display window for viewing selected models and parameter settings. **(C)** Previews of prediction results from multiple GS methods (upper panel) and evaluation results from different metrics (bottom panel). **(D)** Visualization console for viewing prediction results. **(E)** Display window for viewing scatter plots of observed and predicted phenotypes.

The GS analysis panel is composed of five major parts: modeling console, parameter display window, results display window, visualization console, and plot display window. Modeling console consists of G2P console and CV console. From the G2P console, users may select GS methods and corresponding arguments, define indexes of training and test samples, and select evaluation metrics and corresponding arguments (**Figure 4A**). The CV console offers the option of three commonly used CV methods: *k*-fold, holdout, and leave-one-out schemes. Users may also set up the repeat time for CV and proportion of testing set included from the console. As long as all parameters for G2P models and CV methods are set, the display window will exhibit these preset parameters for users to double check (**Figure 4B**). If no further corrections are needed, users may press the execution button to run the GS analysis. It is worth noting that users may select all seven GS methods and eleven evaluation metrics and run G2P prediction and CV evaluation simultaneously to generate a table containing prediction and evaluation results for comparison.

From the results display window, G2P prediction and model evaluation results derived from all GS methods and evaluation metrics selected can be previewed before downloading of the full results (**Figure 4C**). Users may further use the visualization console to visually compare either observed phenotypes and predicted phenotypes or any two sets of predicted phenotypes from any two selected methods (**Figure 4D**). When parameters are set up on the visualization console, a scatter plot depicting the correlation of observed and predicted phenotypes is generated on the plot display window (**Figure 4E**). When IP4GS finishes the analysis of a set of breeding data, users may select the best prediction results from the optimal model to download.

## Discussion

Owing to the rapid advancement of next-generation sequencing, GBTS has greatly reduced the expense of genotyping, making GS-assisted breeding more and more feasible for a growing number of plant species. However, GS analysis requires not only basic bioinformatics skills for data management but also experience in data modeling. The IP4GS platform was developed using the R shiny package, as an interactive, user-friendly web interface, allowing breeders perform GS analysis without the need of bioinformatics skills. However, as with any web-based application, limitations exist. We only integrated seven GS methods commonly used in plant breeding into IP4GS. It is impossible to include all existing methods, especially those ML methods that require intensive computing resources for model training and parameter tuning. The seven methods were selected on the basis of a previously published evaluation of multiple statistical and ML methods, and all seven satisfy three basic criteria (Yan et al., 2021). First, prediction accuracy may not be greatly reduced when the size of the training set is smaller than that of the testing set, since the ratio of training versus test set is usually 1:4 in the seed industry (Yan et al., 2021). Second, model training and CV evaluation may not require too much CPU and memory usage. Third, non-excessive parameters and manual model-tuning are required to properly perform the GS analysis. Another common issue for all web-based applications is the upper size limit of input files uploaded for GS analysis. It is better for users to compile marker sets containing less than 10,000 SNPs, and a population size of smaller than 1,000. Therefore, if a user wants to perform GS analysis of a large dataset or use ML methods consuming intensive computing resource, we do not recommend using IP4GS. Furthermore, IP4GS, as a GS analysis platform, is theoretically applicable to other crops that have successfully applied the GS strategy including rice and wheat. And other species which can provide same format of genotypic and phenotypic data are also applicable but the effectiveness needs further investigation and exploration.

Previously reports indicate that no single GS method outperformed others for all evaluated traits and species. (Heffner et al., 2010; Xiao et al., 2021; Yan et al., 2021). The only solution is to evaluate multiple GS methods and select the optimal one for specific traits and species. Given this need, we integrated multiple GS methods and evaluation metrics so that users may compare results from different predictive models. The seven methods we selected usually generate similar prediction results according to our previous evaluation. Hence, the current version of IP4GS does not include a solution for integration of multi-model prediction results. However, in case of multi-model prediction, we also provided an R script to integrate prediction results from two algorithms. The tool is freely available at https://github.com/furan2019/IP4GSdata.git for users to integrate multi-model prediction results.

## Data availability statement

The original contributions presented in the study are included in the article/supplementary material. Further inquiries can be directed to the corresponding authors.

## Author contributions

SQJ and QC conceived and supervised the project. QC, SQJ, and XFW wrote the manuscript. TL developed the main platform and genomic selection modules of IP4GS. SJ developed the bioinformatics pipeline and analytical modules. RF developed the evaluation modules and visualization modules. All authors contributed to the article and approved the submitted version.
